# Annotations

**Published:** 1890-04-05

**Authors:** 


### Annotations.

**Politicians and Physicians.**

The friends and supporters of Guy's Hospital listened to a broadly-conceived and interesting medical oration from Mr. Gladstone last week. Not only was the speech medical in the sense that it took a survey of medical and surgical history for several centuries, but it dealt to some extent practically with the art of medicine itself. Mr. Gladstone, setting etiquette at defiance, has at least once in his life ventured into the regions of unqualified medical practice. Not only so, but his unhallowed performance proved eminently successful. The story of his flouting of medical tradition he related with much evident satisfaction to the assembled physicians and surgeons at Guy's. He even went further, and ventured to hint that if competent qualified practitioners would occasionally "plough with his heifer," they might enlarge the bounds of their usefulness. The veteran statesman, on a certain occasion when he was testing the temper of his axe's edge, cut his finger somewhat deeply, and there ensued a free flow of blood. Having no pocket-handkerchief, he applied a leaf which happened to be at hand, with the result that not only was the blood promptly staunched, but the healing of the wound took place in about half the usual time. That experiment, with its consequences, was of the nature of a lucky accident rather than of a strictly scientific procedure. Mr. Gladstone says he does not know whether medical men study botany or not as part of their curriculum. The higher grades of medical students do study botany pretty extensively; and one result of their studies is that they avoid making physiological experiments upon either their stomachs or their cut fingers with the leaves or fruit of trees, unless they are able beforehand to ascertain what natural order of plants the particular leaf or fruit they design to use belongs to. For, as is well known, the different natural orders of plants have very different properties, and properties which produce very different physiological effects. It is therefore conceivable that Mr. Gladstone, instead of proving himself a very successful "quack" doctor, might have proved himself the very reverse. The leaf which he applied to his wound might have contained juices which, being absorbed rapidly by the cut blood vessels, would have been dangerous to the injured part, or even to the life. No doubt Mr. Gladstone is a student of botany; and he will do well, if he should cut his finger in future, to consider whether the leaf which he proposes to apply as a remedy belongs to a harmless or a poisonous natural order. Other persons who may read his interesting and lively speech, and incline to follow his example, will also act wisely if they use only the leaves of such plants as they are quite familiar with, and know to be harmless. No doubt some readers will think we are taking rather an alarmist view of very simple facts; but those who have heard the late Sir Robert Christian relate the story of the effect upon himself of his first experiments with Calabar bean will know that plants and their leaves and fruit are by no means always so innocent as they look. Mr. Gladstone heartily congratulated the students of Guy's Hospital on their acquisition of a residential college. Such a college, if wisely administered, cannot but prove of signal use to those who avail themselves of its advantages.

**Canal Boats and their Occupants.**

The outlying regions of lawlessness which continue to exist more or less even in civilized countries, are gradually but surely being brought within the pale of beneficent law in our own land. Canals and canal boats with their population have until quite recent years enjoyed a freedom and indulged in a licence by no means creditable either to themselves or the legislature of their country. The Canal Boats Acts 1877 and 1884 provide for the medical inspection of all boats of this class coming into the Port of London. The Report for 1889 of the working of these Acts has just been issued by the Medical Officer of Health, Dr. W. Collingridge. It shows, among other things, that 624 boats were inspected during the year. The men in charge of the vessels thoroughly understand the meaning and objects of the Acts, and put no difficulties in the way of their being carried out. One of the most prominent evils of the pre-inspection period was overcrowding, and this still exists, but only to a very limited extent. The reason for it is, of course, that the boatmen are too poor to provide houses on shore. Unhappily it is the young children, not the men and women, who are most seriously injured by want of space and bad air. As showing how even "intelligent officials" may help to frustrate the beneficent objects of the very Acts they have to administer, it is pointed out in the report that the 624 boats inspected are registered to carry 2,293 persons, although they are only occupied by 1,531. Whence, then, comes the overcrowding? It is due to the practice of certain authorities who register by the cubic space and not by the accommodation provided. Thus a boat is found to be registered for eight persons simply because it has a certain cubic capacity, whereas actual living accommodation is only provided for three. This is the same as registering a room on land for eight persons which is only capable of accommodating three, simply because it happens to be in a large house and a wide street. The problem of furnishing permanent officials with common sense and reasonably kind feelings is one of the most difficult which modern civilization has to solve. It is satisfactory to find the report stating that the boats are generally well kept. It is still more encouraging to learn that the masters look favourably upon the inspectors, as enabling them to obtain needed alterations and repairs. The "family" boat is, it is said, fast disappearing, and this, as can easily be imagined, is fraught with the most important moral and physical advantages. During the whole year not a single case of infectious disease was met with in any of the boats. Other points are brought out in the brief but excellent report of Dr. Collingridge, and it is stated generally that the experience of the working of the Canal Boats Acts in the Port of London is on the whole favourable, and that a slow improvement in the morality and condition of the canal population is certainly to be observed. The Acts are being applied very gradually; and it is probably this gradual application that reconciles the boatmen and their families to their changed circumstances.

**The Fasting Man.**

England is the very country in which such experiments as Giovanni Succi is now making at the Royal Aquarium should be carried out. We are so sceptical about the wonderful and so impatient with the unpractical, that we are quite sure to find out any imposture if any be attempted. Succi says he is going to fast forty days. As we write the first fortnight of his period has almost passed away; and the experimenter is said to be both well and cheerful, and to have entirely got rid of his craving for food. He drinks water freely, and mixes with it from time to time small portions of his wonderful elixir. Many people doubt whether it is worth while to find out how long a man can live without food. Certainly it is; if not from the purely scientific, at least from the practical point of view. There are stories of long fasts related in the Bible, and as many *a priori* reasoners are quite ready to declare the impossibility of such facts on the authority of their own inner consciousness, it is important to make clear that they are at least possible. The questioners will then have learnt this valuable principle—that historical testimony may be as valid as *a priori* reasoning. Moreover, it often happens at sea, in barbarous countries, and elsewhere, that human beings are deprived of food for long periods. Such deprivation, besides the actual debility which it induces, gives rise to profound mental depression and anxiety. These purely mental conditions probably often cause starving men and women to die hours or even days sooner than they would were hope to be preserved strong in the breast. The tendency of experiments like Succi's is to increase the faith of all men in the staying power of the human creature. Such an increase of faith, with its power of inspiring hope in urgent straits, may save many valuable lives. Although, therefore, the performance may have been undertaken with very mixed or even unworthy motives, and may be held by sober people to effect nothing more than the gratification of mere vulgar curiosity, yet has it its better side. If Succi succeeds in fasting forty days, or anything like forty days, he will give us all a deeper appreciation of the staying power of the human organism, and more faith in our own resolution to brave the worst if necessity should arise.

